# Clinical value of diascopy and other non-invasive techniques on differential diagnosis algorithms of oral pigmentations: A systematic review

**DOI:** 10.4317/jced.53005

**Published:** 2016-10-01

**Authors:** Daniel Pérez-López, Maite Pena-Cristóbal, Eva-María Otero-Rey, Inmaculada Tomás, Andrés Blanco-Carrión

**Affiliations:** 1 PhD Student, Facultade de Medicina e Odontoloxía, Departamento de Estomatoloxía, Universidade de Santiago de Compostela, Spain; 2 Postgraduate Student, Facultade de Medicina e Odontoloxía, Departamento de Estomatoloxía, Universidade de Santiago de Compostela, Spain; 3 PhD, Facultade de Medicina e Odontoloxía, Departamento de Estomatoloxía, Universidade de Santiago de Compostela, Spain; 4 Senior Lecturer, Oral Sciences Research Group, Facultade de Medicina e Odontoloxía, Departamento de Estomatoloxía, Universidade de Santiago de Compostela, Spain; 5 Senior Lecturer, GI-1319, Facultade de Medicina e Odontoloxía, Departamento de Estomatoloxía, Universidade de Santiago de Compostela, Spain

## Abstract

**Objectives:**

To determine the diagnostic value of diascopy and other non-invasive clinical aids on recent differential diagnosis algorithms of oral mucosal pigmentations affecting subjects of any age.

**Material and Methods:**

Data Sources: this systematic review was conducted by searching PubMed, Scopus, Dentistry & Oral Sciences Source and the Cochrane Library (2000-2015); Study Selection: two reviewers independently selected all types of English articles describing differential diagnosis algorithms of oral pigmentations and checked the references of finally included papers; Data Extraction: one reviewer performed the data extraction and quality assessment based on previously defined fields while the other reviewer checked their validity.

**Results:**

Data Synthesis: eight narrative reviews and one single case report met the inclusion criteria. Diascopy was used on six algorithms (66.67%) and X-ray was included once (11.11%; 44.44% with text mentions); these were considered helpful tools in the diagnosis of intravascular and exogenous pigmentations, respectively. Surface rubbing was described once in the text (11.11%).

**Conclusions:**

Diascopy was the most applied method followed by X-ray and surface rubbing. The limited scope of these procedures only makes them useful when a positive result is obtained, turning biopsy into the most recommended technique when diagnosis cannot be established on clinical grounds alone.

** Key words:**Algorithm, differential diagnosis, flow chart, oral mucosa, oral pigmentation, systematic review.

## Introduction

Oral mucosal pigmentations are relatively common in daily dental practice and usually mean a diagnostic challenge for clinicians (1-3).

In this sense, diascopy has been proposed as a possible diagnostic tool for this type of conditions, being defined as a procedure of removing the camouflaging effect of congested blood to reveal the true colour of underlying lesions (4). This is done by means of a glass or plastic diascope, usually a microscopic slide, pressed against a cutaneous or mucous lesion (4-6). Its characteristic blanching effect is due to the phenomenon of blood dissipating intravascularly under compression, giving the tissue a pale appearance (4,7,8). Even though dermatologists regularly use epiluminescence microscopy in the early diagnosis of malignant melanoma and pigmented skin lesions, magnified or unmagnified diascopy is sometimes applied on large cutaneous pigmentations (5,6,9-12). In dentistry, its most common application consists of obtaining a positive result for blanchability to potentially identify the intravascular nature of oral pigmented lesions; however, not all intravascular conditions seem to comply with this rule (4). This is of utmost importance for an accurate diagnosis and the appropriate management of oral pigmentations. In view of the lack of any study that has methodologically assessed the current clinical value of diascopy and other non-invasive clinical aids on this kind of lesions, this new systematic review has been conducted to provide scientific evidence on this field.

-The following objectives were addressed:

•Primary: to determine the current diagnostic value of diascopy on the differential diagnosis of oral mucosal pigmentations, all types of recently published articles that described a differential diagnosis algorithm about such lesions in which this diagnostic technique was present were reviewed, against those that did not use it, aimed at subjects of any age affected by these oral conditions.

•Secondary: to determine the diagnostic value of other non-invasive clinical aids on the previously mentioned differential diagnosis flow charts, as well as the most recommended method to reach a definitive diagnosis.

## Material and Methods

- Protocol

This systematic review was conducted according to a previously established protocol. Likewise, the PRISMA Statement recommended items were addressed whenever possible (13).

- Eligibility criteria

Types of studies: all kind of recently published articles describing differential diagnosis flow charts of oral mucosal pigmentations, including diascopy or not as a diagnostic step were considered. Only the most mentioned lesions in several related review articles were taken into account (2,3,14,15), to which haemangiolymphangioma, peripheral giant cell granuloma and thrombus were added based on the authors´ experience ( Table 1). Furthermore, non-English papers were excluded (16) and a 15-year period was established to conduct the review.

**Table 1:**
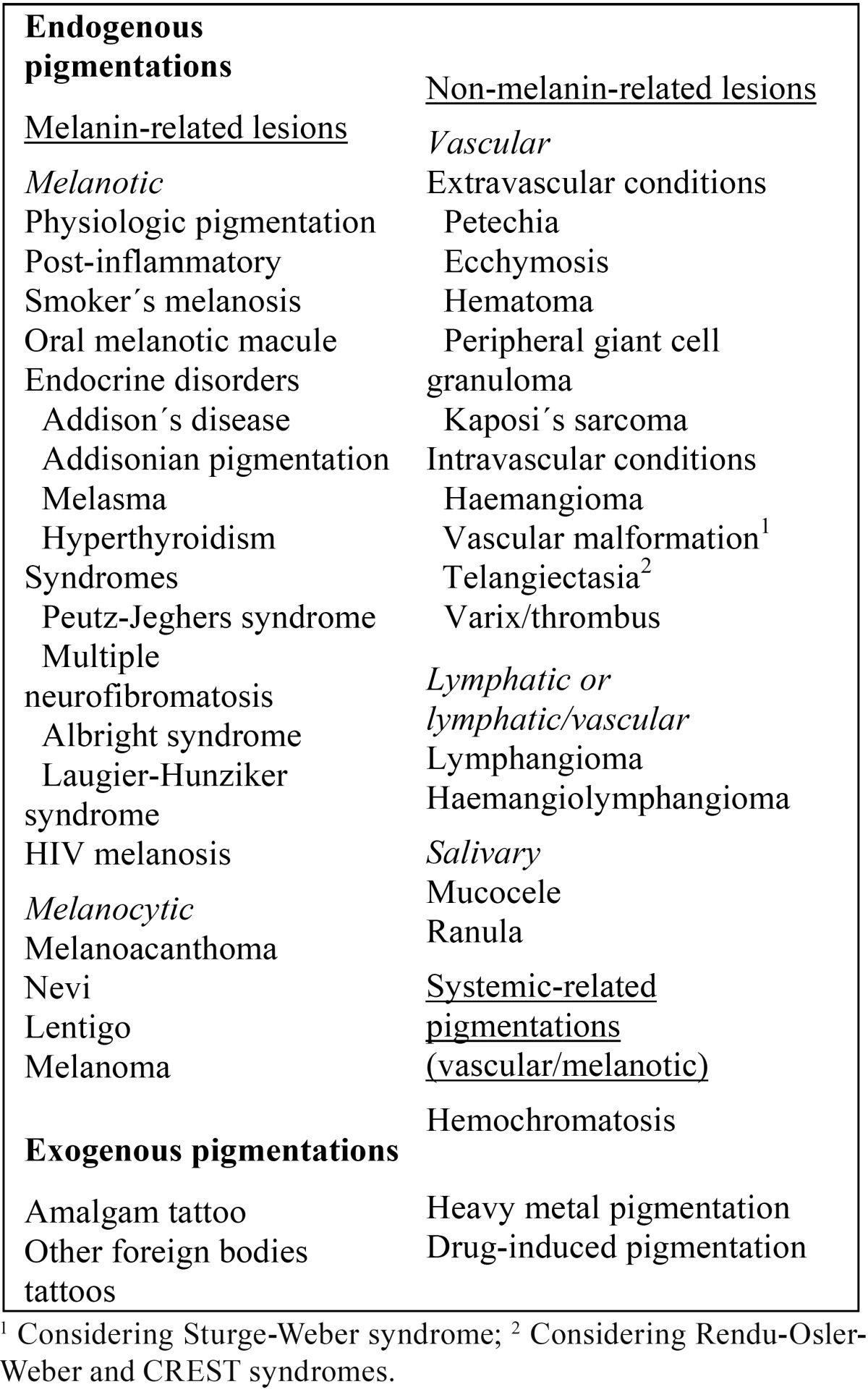
Oral pigmentations considered on study screening.

Types of participants: subjects of any age affected by oral mucosal pigmentations.

Types of intervention: application of diascopy or other non-invasive clinical aids on oral mucosal pigmentations.

Types of comparator: no application of diascopy or other non-invasive clinical aids on such lesions.

Types of outcome measures: 1. Primary: the diagnostic value of diascopy on current differential diagnosis protocols of oral mucosal pigmentations; 2. Secondary: the diagnostic value of other non-invasive clinical techniques on these protocols and the most recommended method to achieve a definitive diagnosis.

- Information sources

Four electronic databases were searched: PubMed (National Center for Biotechnology Information, U.S. National Library of Medicine, National Institutes of Health), Dentistry & Oral Sciences Source, DOSS (EBSCO Host), and the Cochrane Library (Wiley Online Library) and Scopus (Elsevier). A time filter was imposed from 1 January 2000 to 25 May 2015 and all the databases were researched up to 1 June 2015 (a total period of 15 years and five months). No language filter was imposed in this phase. Reference checking of finally included articles was also performed. Additionally, authors of these finally selected papers were contacted to clarify doubtful information, absent data and confirm the extracted evidence. The search protocol was developed and conducted by one of the reviewers (DPL), once it was validated by the group.

- Search 

The following search terms were used to search the four databases: oral cavity, oral mucosa, pigmented, pigmentation, pigmentations, vascular, discoloration, discolorations, discolouration, discolourations, hyperpigmentation, hyperpigmentations, lesion, lesions, diagnosis, flow chart, protocol, differential diagnosis, algorithm, and guide. The search strategy for each database was previously specified in the systematic review protocol.

- Study selection 

Once the articles were retrieved from each of the four databases and the duplicates were dismissed by one of the reviewers (DPL), the eligibility assessment of all the references was independently carried out by two reviewers (DPL and MPC). As a first step, the titles and abstracts were screened by language and inclusion criteria. Then, the full texts of potentially relevant studies were examined for inclusion criteria compliance. Reference checking of finally included studies was first performed by title; if the corresponding abstracts were considered suitable, the full texts were examined. When titles and abstracts did not provide enough information to make a decision or the abstracts were not available but the titles were considered suspicious of being related to the purposes of the review, the respective full articles were assessed. Reasons for exclusion were stated at each stage, except for title selection during reference checking. Disagreements between reviewers were solved by consensus at each step. If the two reviewers did not agree, a third investigator (IT) was contacted.

- Data collection process

A data extraction sheet based on *a priori* established data items was developed and accordingly modified after pilot-testing it on three of the finally included studies. On this occasion, only one of the reviewers (DPL) extracted the data from the corresponding studies while the other reviewer (MPC) checked its validity. Again, disagreements were solved by consensus. If no agreement could be reached, a third investigator (IT) was consulted.

As stated, authors of the finally included articles were contacted to obtain ambiguous or absent data, as well as to confirm the performed data extraction.

- Data items

Information was extracted from each included study on.

1) Study design: justification, aims, type of study according to previously reported classification (17), type and language of cited documents, and funding.

2) Participants: features of the population for which the differential diagnosis protocol was developed, type of included lesions, and features of case reports, if present.

3) Intervention/comparator: main aspect that determines the first step on the differential diagnosis protocol; presence of diascopy, at which level and type of lesions; and presence of other clinical diagnostic techniques, at which level and type of lesions.

4) Outcomes: diagnostic value of diascopy and other non-invasive clinical techniques applied on the included protocols, as well as the most recommended method for reaching a definitive diagnosis, and other conclusions.

As already reported, the data extraction sheet was developed *a priori*, but three additional items were added after reading the finally included articles. It was decided to include the type and language of the documents cited on finally included papers to provide information about what kind of data they were based on. Likewise, the assessment of the main aspect or procedure that determined the first step on each of the differential diagnosis protocols was introduced since it was considered to be a very useful contribution to daily dental practice.

- Quality assessment

Since the finally included studies were expected to mainly consist of narrative reviews, risk of bias assessment was not considered. For this reason, their “quality” was assessed through a self-designed checklist based on six parameters that were considered important for their clinical application. Based on the authors’ clinical experience, three features related to the algorithm *per se* and another one focused on the article text were initially proposed for the quality appraisal: one classificatory aspect per step (to allow establishing a hierarchical diagnostic process), self-explanation of each step (clearly described clinical aspects or techniques to ease its implementation), presence of distinctly specified lesions (to clarify which types of lesions clinicians should mainly consider with each protocol) and description of outstanding malignancies in text (to alert clinicians to base the differential diagnosis on their exclusion). This checklist was modified after completely reading the articles selected by screening titles and abstracts, with two more text parameters added: thorough explanation of the algorithm (to reinforce its understanding and solve any doubt from its isolated assessment) and clearly stated algorithm limitations (to become aware of its scope).

As in the data collection process, the quality assessment was performed by one of the reviewers (DPL), while the other reviewer (MPC) checked its validity. In the case of several flow charts, only algorithms based on oral pigmentations were considered. Disagreements were solved by consensus. If no agreement could be reached, a third investigator (IT) took part.

- Summary measures and synthesis of results

A descriptive data analysis was performed. The following aspects were considered: the percentage of studies applying diascopy or other non-invasive clinical techniques (either in protocols or in protocol/text combination), the most recommended method for a definitive diagnosis and its percentage of use (regardless of its place of mention), and the different main aspects or procedures that determined the first step in the several algorithms and their prevalence (either individually or coupled with others) ( Table 2).

**Table 2:**
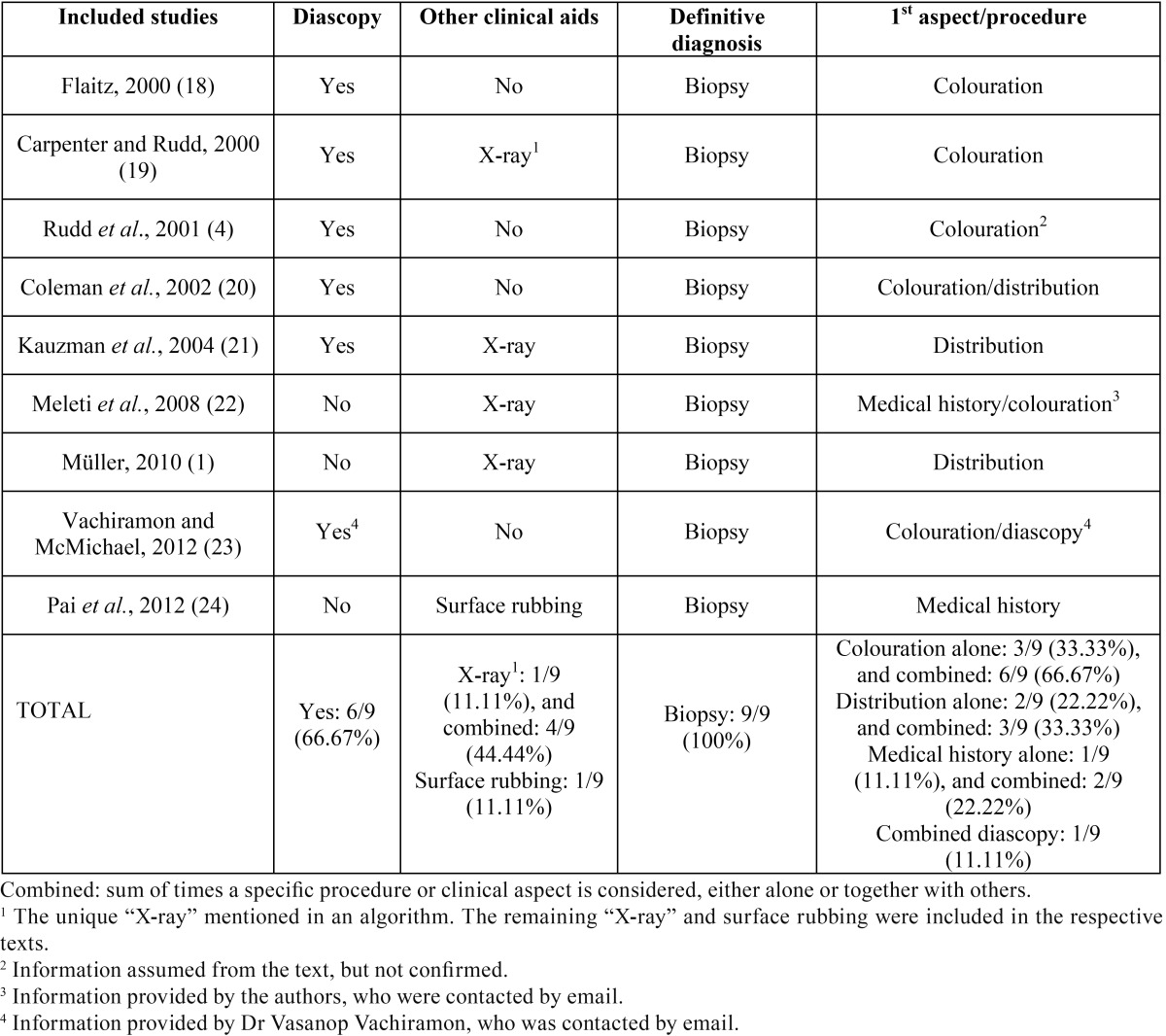
Results of individual studies and synthesis, in chronological order.

## Results

- Study selection

**Figure 1:**
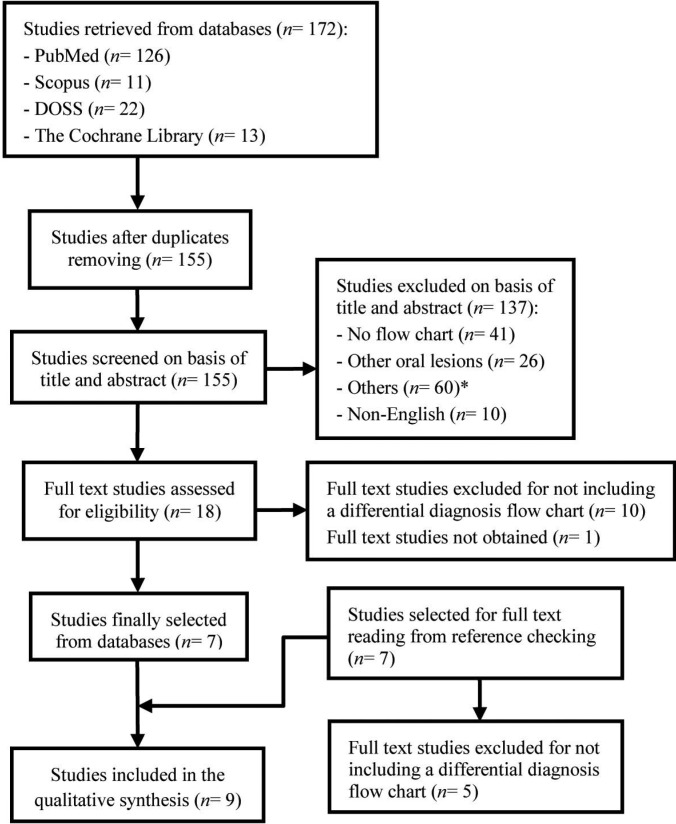
Flow of information through the different phases of the systematic review. *Comprising studies of other medical disciplines, congress abstracts, articles related to caries or not focused on differential diagnosis (regardless of the type of lesions assessed), lesions beyond the limits of the oral cavity, and miscellaneous.

A total of nine studies were finally included in the review. The searches of PubMed, Scopus, DOSS and the Cochrane Library databases provided a total of 172 studies. After adjusting for duplicates, 155 remained. Of these, 137 studies were discarded after reviewing their titles and abstracts since it was considered that they did not meet the inclusion criteria. The exclusion criteria in this phase were classified into four groups: group 1 (n= 41), involving studies that mentioned the previously reported included lesions without evidence of a differential diagnosis flow chart; group 2 (n= 26), in relation to studies that described other oral lesions (distinct from caries); group 3 (n= 60), comprising studies of other medical disciplines, congress abstracts, articles related to caries or not focused on differential diagnosis (regardless of the type of lesions assessed), lesions beyond the limits of the oral cavity, and miscellaneous; and group 4 (n= 10), concerning non-English studies. Thus, 18 studies were selected for the full texts to be read. It appeared that ten studies did not meet the inclusion criteria and another one could not be retrieved, so seven articles were included in the systematic review from the database electronic search. Additionally, seven studies were also considered for full text reading by checking the references of the seven studies mentioned above; five of them were excluded for not providing a differential diagnosis flow chart. Therefore, a total of nine studies were finally included in our review (Fig. 1).

Eight authors of the included articles were contacted by email for further information, mainly related to the funding source, population features and the main aspect or procedure that determined the first step in the differential diagnosis algorithms. Likewise, the same authors were contacted to verify the accuracy of the extracted data by sending them a copy of the respective draft chart by email. All authors responded, with one exception that provided the first-stage information without subsequently confirming the data extraction. The whole process was performed by one of the reviewers (DPL).

- Study characteristics ( Table 3,  Table 3 continue,  Table 3 continue-1)

**Table 3:**
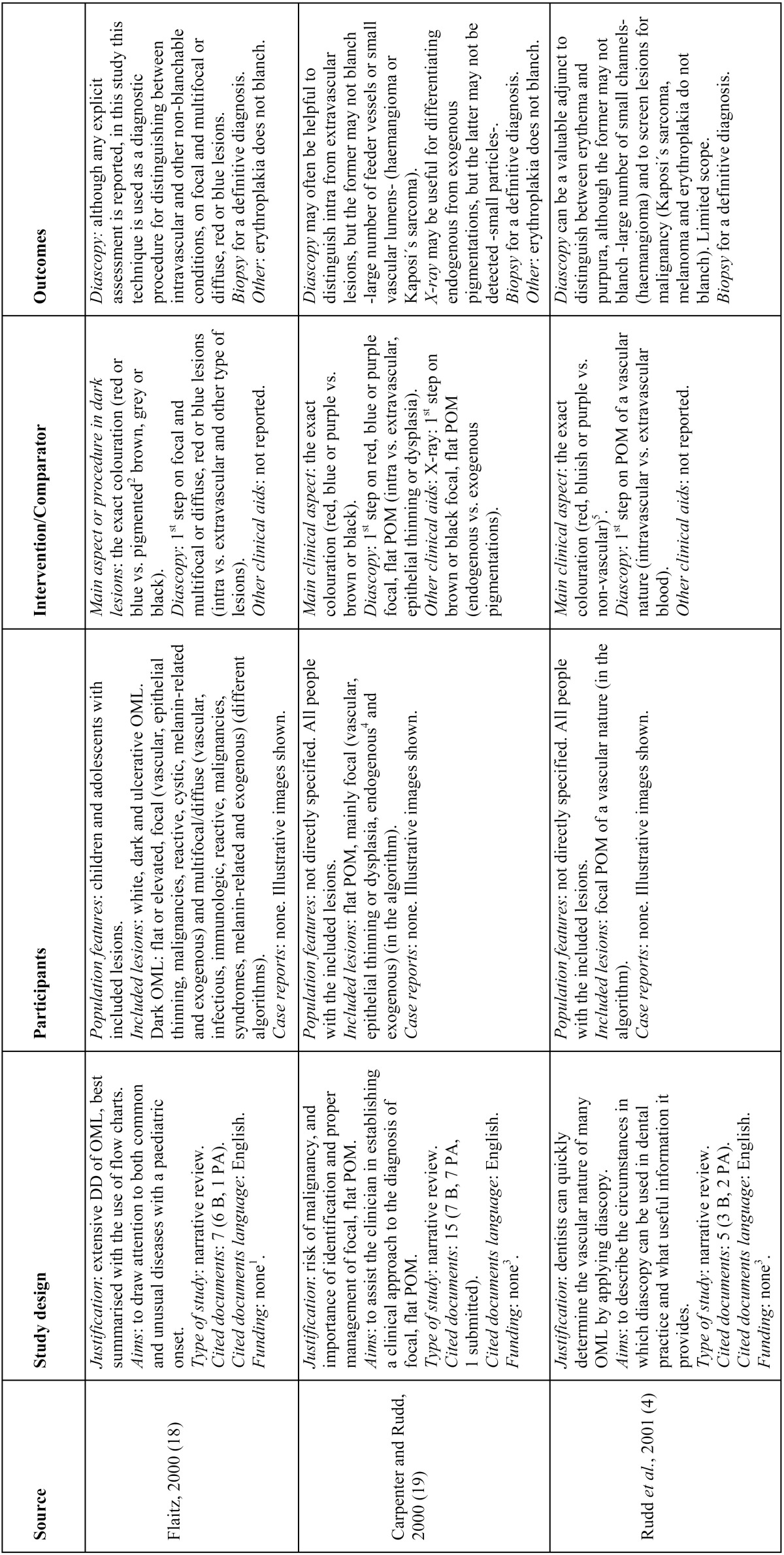
Characteristics of the included studies, in chronological order.

**Table 3 continue:**
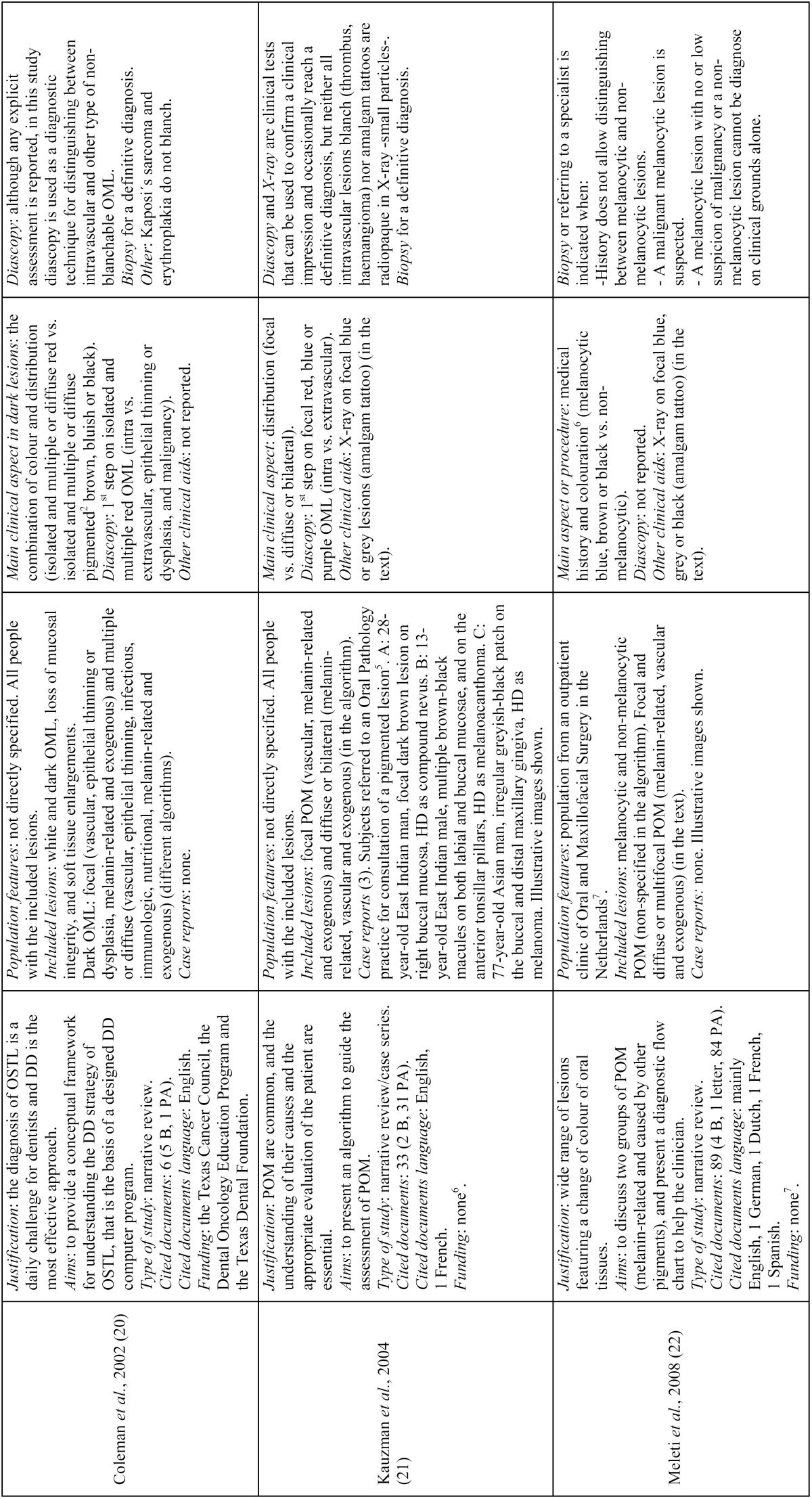
Characteristics of the included studies, in chronological order.

**Table 3 continue-1:**
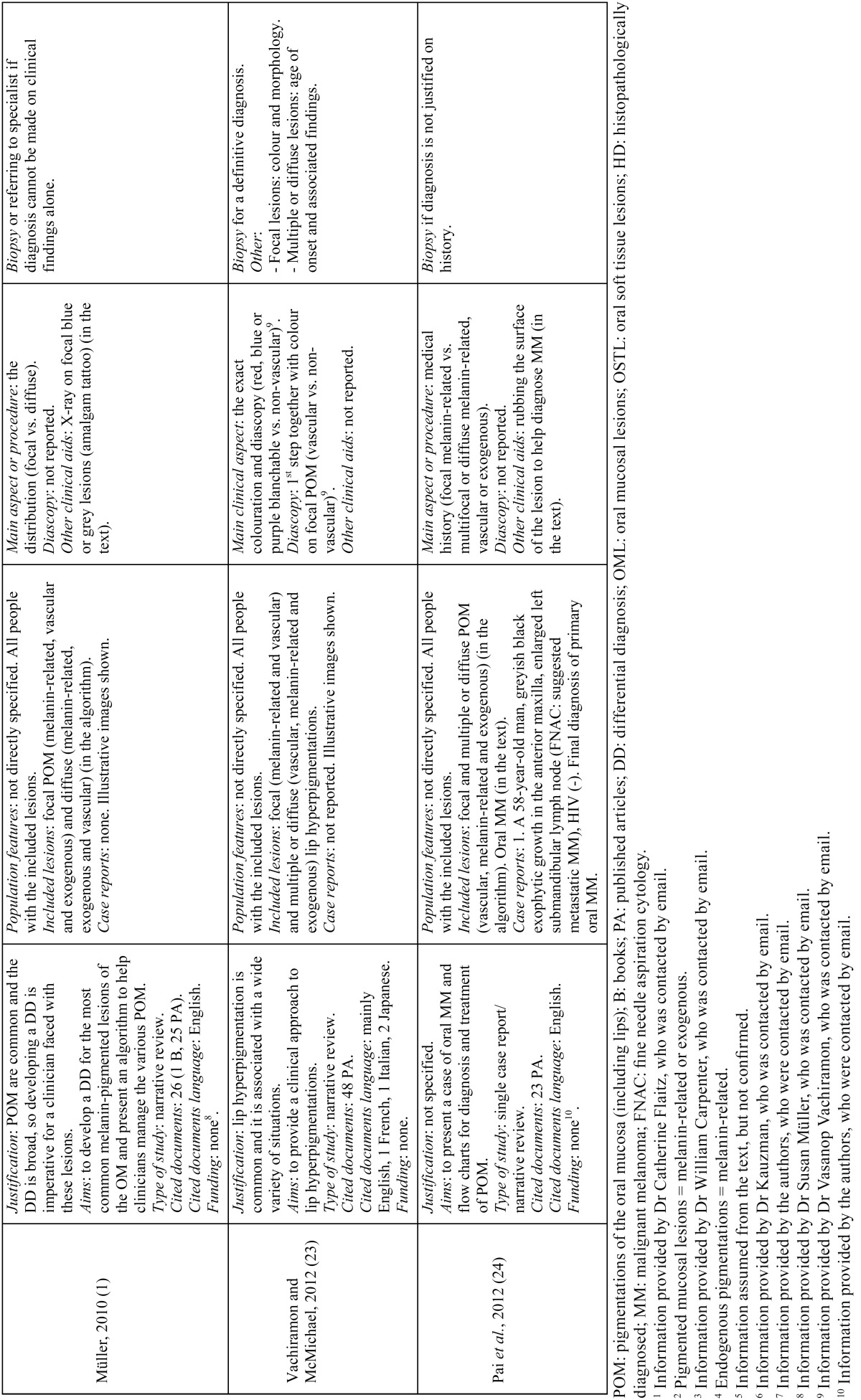
Characteristics of the included studies, in chronological order.

•Study design

The nine studies finally selected for the qualitative assessment consisted of eight narrative reviews (1,4,18-23) and one single case report (24). One narrative review was followed by a case series of three melanin-related oral pigmentations (21) and the oral melanoma case report was followed by a narrative review mainly based on this entity (24). Almost all of the included studies arose from the large variety of mucosal pigmentations that may appear in the oral cavity and their challenging differential diagnosis, with the main purpose of helping clinicians identify and manage them. Within these studies, the greatest part of the cited documents was in English and mainly comprised of published articles on the global calculation; nonetheless, some papers individually showed the same or greater number of book citations than published articles (4,18-20). Only one study had a funding source (20).

•Participants

With the exception of two articles (18,22) that were respectively focused on children and adolescents and on subjects from an outpatient clinic of Oral and Maxillofacial Surgery, the remaining papers (1,4,19-21,23,24) did not specify the features of the population beneficiary of the differential diagnosis protocols beyond subjects affected by the corresponding included oral lesions.

Lesions included in the algorithms varied from just oral pigmentations, either focal or multifocal/diffuse, melanin or non-melanin related (1,4,19,21-24), to oral pigmentations coupled with white lesions, ulcerative conditions and tissue enlargements (18,20).

As already mentioned, one article reported three cases of oral pigmentations histopathologically diagnosed as compound nevus, melanoacanthoma and melanoma, in a 28-year-old East Indian man, a 13-year-old East Indian male and a 77-year-old Asian man, respectively (21). Additionally, another article reported a case of malignant melanoma in a 58-year-old man diagnosed through its clinical appearance, fine needle aspiration cytology of the left submandibular lymph node, orthopantomograph, computed tomography and a complete haemogram (24).

•Intervention/comparator

Diascopy was used in six protocols (4,18-21,23), while only one algorithm reported the use of X-ray as a non-invasive clinical aid (19); nonetheless, the use of X-ray was mentioned more in text (1,21,22). Likewise, surface rubbing was mentioned once in the text in the diagnosis of oral malignant melanoma (24). Colour determination was the most frequently described first step amongst algorithms, either alone or combined (4,18-20,22,23).

•Outcomes 

•Primary

Three articles stated briefly the diagnostic value of diascopy (4,19,21), while the other three studies that included this technique in their algorithms did not state its value anywhere (18,20,23).

•Secondary

Two articles briefly described the clinical value of X-ray (19,21), while the other two that mentioned its use did not state its value (1,22). Biopsy was recommended everywhere to reach a definitive diagnosis (1,4,18-24).

- Quality assessment ( Table 4)

**Table 4:**
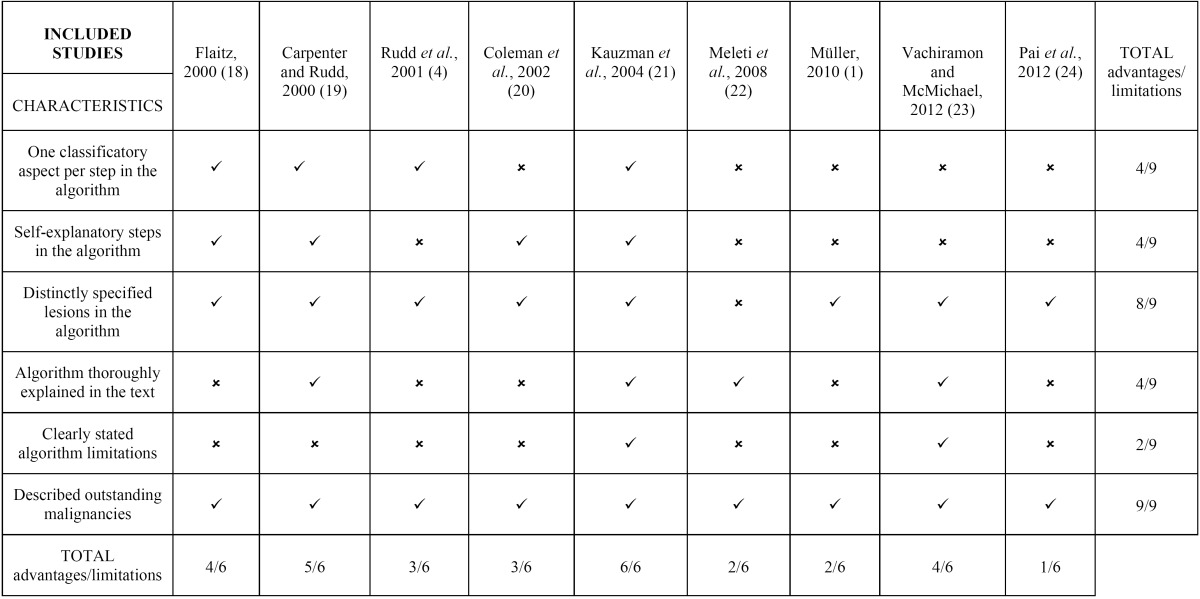
Quality assessment of included studies, in chronological order.

Only one study met all of the parameters assessed (21). One study met five of them (19) and the other two fulfilled four (18,23). The remaining articles complied with three (4,20), two (1,22) or one aspect (24).

All of the studies reported outstanding malignancies (1,4,18-24) and almost all of them included distinctly specified lesions in their algorithms (1,4,18-21,23,24). The less incorporated parameter was the statement of algorithm limitations (21,23).

- Results of individual studies and synthesis ( Table 2)

As has already been commented upon, the main aspect or procedure that determined the first step in the differential diagnosis protocols was added to the results related to the already known review outcomes.

Diascopy was used on six of the nine included protocols (66.67%), being mainly applied to red, blue or purple, focal and multifocal/diffuse oral pigmentations, to distinguish between intravascular conditions and other type of lesions (4,18-21,23). Of these, only three studies briefly stated the clinical value of diascopy, considering it a helpful and valuable tool for the previously reported objective and to screen lesions for malignancy, confirming a clinical impression and sometimes reaching a definitive diagnosis. However, it was stated that not all intravascular conditions blanch under pressure (4,19,21).

Only one of the nine algorithms (11.11%) reported the use of X-ray on brown or black focal lesions (19). Nonetheless, in the text of the other three articles (44.44% with the previous one), the use of this technique was described in the diagnosis of isolated blue or grey lesions with the same purpose (1,21,22). Similar to before, only two articles punctually assessed the clinical value of X-ray, considering it a helpful tool for differentiating melanin-related conditions from exogenous pigmentations, confirming a clinical impression and sometimes reaching a definitive diagnosis. Nevertheless, it was reported that exogenous pigmentations may not be detected by X-ray (19,21).

Likewise, one article (11.11%) described in its text the procedure of surface rubbing in the diagnosis of oral malignant melanoma, without evaluating its diagnostic value (24).

Biopsy was recommended without exception to reach a definitive diagnosis when this cannot be made on clinical grounds alone (100%) (1,4,18-24).

Colour determination was the main clinical aspect that determined the first step in three of the nine (33.33%) included differential diagnosis protocols (4,18,19). The distribution of lesions was the main clinical aspect in two of them (22.22%) (1,21), the combination of colour and distribution in one (11.11%) (20), colour and medical history in another (11.11%) (22) and colour and diascopy in the other (11.11%) (23). The remaining protocol seemed to be based on medical history alone, without mentioning any specific clinical aspect (24). Results from combined data (sum of times in which a clinical aspect or procedure was applied as a first step, either alone or together with other aspects or techniques) were: colouration (6/9, 66.67%), distribution (3/9, 33.33%), medical history (2/9, 22.22%) and diascopy (1/9, 11.11%).

## Discussion

Many narrative reviews have been published describing large sets of oral pigmented lesions without providing a differential diagnosis flow chart (2,3,14,15,25-31). Only some of them have considered vascular lesions to be true oral pigmentations (3,14,25,27,30,31) and the use of diascopy has been proposed in these cases with the same aforementioned purposes (3,14,25,27). Likewise, this technique has been coupled with the so-called “head lowering manoeuvre with abdominal compression” in the diagnosis of eight oral capillary haemangiomas, considering clinical appearance combined with a positive result in at least one of the previous procedures a sufficiently reliable method for their identification and treatment (32). Despite the lack of evidence in this regard, the reported positive result for blanchability in Kaposi´s sarcoma in one of our included studies (19) seems not to be supported by histological data since red blood cells extravasation is an almost constant feature in all stages of the lesion and in almost all of its microscopic variants, although it may not be as evident in early patches (33-37); nonetheless, a negative blanching result even in these initial lesions has been stated (38). Colour interpretation is a subjective procedure; small differences may be difficult to notice and the final colouration is conditioned by the amount and location of the pigment within the mucosa (1,21). In spite of the frequent use of this visual sign, there is some heterogeneity amongst algorithms and texts in this regard; for instance, blue may be considered to represent either a vascular lesion, a foreign-body tattoo or a melanin-related condition (19-21,23). Although clinicians should know that some colours are more related to vascular or melanin conditions, based on the frequent colour superimposition, we recommend using diascopy on all dark oral pigmentations, mainly focal and regardless of colour, when the possibility of a vascular lesion is being considered and the technique can be applied due to location. Only a complete or significant positive result for blanchability will be useful in clinical practice, since a semi- or non-blanchable result will make the clinician feel unsure of the diagnosis and a biopsy should be considered.

In relation to X-ray, amalgam tattoo is more present on oral pigmentation-related reviews and the application of this technique has been well-mentioned on its differential diagnosis (3,14,15,26,30,31); nonetheless, it has been reported that fewer than 25% of these entities will be seen as radiopaque on radiographs (26). For the same reported reasons, we recommend using X-ray on all focal flat or slightly raised blue, brown, grey or black oral mucosal lesions, trying to rule out an exogenous pigmentation. If a negative result is obtained, a biopsy should be performed if the possibility of an oral malignant melanoma cannot be dismissed on clinical findings alone.

Despite the frequent presence of oral melanoma in many narrative reviews, surface rubbing is not described as a clinical tool on its diagnosis (2,3,14,15,25-31). This procedure was first applied on 13 subjects with a clinical suspicion of oral primary melanoma and a positive result was achieved on 11 of the finally 13 histopathologically diagnosed melanomas (39). Since then, some authors have reported its use (40-44); nevertheless, it seems that this technique has not been broadly implemented in the literature and its value has been questioned (45).

As expected, histopathology assessment has elsewhere been considered the gold standard method for reaching a definitive diagnosis in oral pigmented lesions (2,3,14,15,25-28,30,31).

Our study has several limitations. Regarding the study and review level, the search retrieved results were screened for just including English-language publications and only studies published in the last 15 years were considered. Additionally, the set of included lesions may have obviated some conditions that could have led us to other diagnostic flow diagrams. Likewise, the performed title/abstract search restriction was applied to best focus on potentially eligible papers since it was assumed that articles without including any of those words would not be selected. In relation to the quality assessment, the evaluated parameters were based more on the applicability of the diagnostic flows than on their quality, so the punctuations are not directly associated with the latter. Finally, the Oxford Centre for Evidence-Based Medicine does not currently consider narrative reviews or single case reports on its Levels of Evidence Table (46). In this sense, the articles included in this review would probably be classified into Level 5 regarding the rather well-recognised evidence weakness of these study types. Moreover, the high presence of case reports, case series and narrative reviews cited in them and not directly related to the previously assessed diagnostic techniques makes establishing a definitive evidence level demanding. Nonetheless, in this particular case, better evidence about the previously addressed topics is not expected to appear. Taking this into account, as well as the corresponding benefits and harms of the reported procedures, the recommendations stated in this review are considered to be evidence-supported enough for their implementation in daily dental practice.

Based on this evidence, it was finally concluded that diascopy was the most applied diagnostic technique on the recent differential diagnosis algorithms of oral pigmented lesions, followed by X-ray and surface rubbing. The limited scope of these techniques only makes them useful when a positive result is obtained and turns biopsy into the most recommended procedure when a diagnosis cannot be made on clinical grounds alone.

Interested authors are encouraged to clearly state all of the clinical techniques applied on their case report and case series studies, as well as the respective obtained results, also providing a histopathological diagnosis. Such information will allow investigators and clinicians to accurately assess the diagnostic value and limits of those applied procedures and their relation to different types of lesions and their variants.
